# Effect of Crown Design, Cement Type and Margin Depth on the Removal of Cement Remnants Around Single Implant‐Supported Restorations. An In‐Vitro Study

**DOI:** 10.1111/clr.70129

**Published:** 2026-04-12

**Authors:** Shaza Bishti, Christiane Apeldorn, Anne Rittich, Stefan Wolfart, Taşkın Tuna

**Affiliations:** ^1^ Department of Prosthodontics and Biomaterials, Centre for Implantology Uniklinik RWTH Aachen Aachen Germany

**Keywords:** cement, cement remnants, crown design, implant‐supported, margin depth

## Abstract

**Objectives:**

To assess the influence of crown design, cement type, and margin depth on the removal efficiency of residual cement around single implant‐supported restorations.

**Methods:**

Twelve mandibular models with a missing first molar were fabricated and provided with single implant analogs (centrally/distally placed) and two different crown designs (non‐cantilever/cantilever). Three of each design had either a superficial (SM = −1 mm) or a deep (DM = −2 mm) submucosal margin. Crown restorations were made from CAD/CAM titanium‐base abutments and monolithic zirconia (*n* = 120). Three different cements (glass ionomer [GI], polycarboxylate [PC], dual‐curing resin cement [RC]) were used in a standardized way for crown cementation. Ten dentists removed excess cement using metal scalers. The volumetric values of residual cement were calculated (mm^3^). Statistical analyses assessed the effects of the investigated variables on residual cement volume (*p* < 0.05).

**Results:**

The overall analysis showed no significant differences in the volume of cement remnants between both crown designs (non‐cantilever: 0.42 ± 0.96 mm^3^; cantilever: 0.52 ± 0.98 mm^3^; *p* > 0.05). For crown margin depth, a significantly higher cement volume for deeper margins (SM: 0.28 ± 0.62 mm^3^; DM: 0.68 ± 1.19 mm^3^; *p* < 0.05) was found. The highest volume of cement remnants was found in the non‐cantilever_DM_RC (1.19 ± 1.83 mm^3^), whereas the lowest value was found in non‐cantilever_SM_GI and cantilever_SM_GI (0.00 ± 0.00 mm^3^). While crown design alone showed no statistically significant effect, its interaction with cement type was associated with improved cement removal in the cantilever group.

**Conclusion:**

Crown margin depth emerged as the dominant factor influencing cement removal. Although cement type also affected residual cement, with glass ionomer showing lower values overall, crown design did not provide a consistent advantage.

## 1 Introduction

Dental implants are widely regarded as a reliable and predictable solution for the replacement of missing teeth (Duong et al. 2022; Pjetursson and Heimisdottir 2018; Sartoretto et al. 2023). Despite their high success and survival rates, maintaining peri‐implant health remains a significant clinical concern (Schwarz and Ramanauskaite 2022). Peri‐implant mucositis and peri‐implantitis are among the main factors that can affect the longevity of implants, with biofilm recognised as the major cause of both conditions (Lindhe and Meyle 2008). A recent systematic review and meta‐analysis reported a prevalence of approximately 63% for peri‐implant mucositis and 25% for peri‐implantitis, based on the 2017 World Workshop criteria, emphasizing their global reference (Derks and Tomasi 2015; Lee et al. 2017; Reis et al. 2025). Given the unpredictable outcomes of current treatment approaches for peri‐implantitis, emphasis has shifted toward preventive care including consistent monitoring, meticulous maintenance by clinicians, and effective oral hygiene practices by patients as supported by recent consensus reports and clinical practice guidelines (Armitage and Xenoudi 2016; Berglundh et al. 2018; Herrera et al. 2023).

The anatomical and structural differences between teeth and implants further complicate hygiene accessibility, particularly in interproximal areas. Factors such as implant diameter, three‐dimensional implant position, and restoration contour may promote the formation of niches that are difficult to access and favor microbial colonization (Saleh et al. 2022). Overcontoured restorations—characterized by convex emergence profiles and emergence angles commonly reported to exceed 30°—have been associated with limited access for oral hygiene and may be linked to an increased prevalence of peri‐implant complications, regardless of whether the restorations are screw‐ or cement‐retained (Schwarz et al. 2018; Sirirattanagool et al. 2025). These features can hinder plaque removal, facilitate biofilm accumulation, and contribute to the onset of peri‐implant diseases (Atieh et al. 2023; Izzetti et al. 2025; Sancho‐Puchades et al. 2017; Sirirattanagool et al. 2025).

Cemented implant‐supported restorations remain widely used in clinical practice due to their favorable esthetics, ease of fabrication and their similarity to conventional fixed restorations (Chee and Jivraj 2006; Lee et al. 2010; Michalakis et al. 2003). They allow flexibility in achieving optimal crown contour, emergence profile, and occlusal morphology, without compromising the ceramic surface through an access hole, as is necessary in screw‐retained restorations (Chee and Jivraj 2006; Lee et al. 2010; Michalakis et al. 2003). With proper diagnostics, backward planning, and the selection of appropriate prosthetic abutments, screw‐retained restorations can achieve many of the advantages traditionally associated with cemented crowns. The main benefit that remains unique to screw‐retained restorations is their retrievability. Additionally, cemented restorations carry a higher biological risk than screw‐retained ones, primarily due to submucosal retention of excess cement. Therefore, when cemented restorations are necessary, the crown margin should be placed at the mucosal level to facilitate cement removal, and care should be taken to ensure healthy, stable peri‐implant soft tissue conditions. Consequently, cemented crowns should generally be reserved for specific clinical situations, such as compromised esthetics requiring buccal screw access (Staubli et al. 2017). It has been reported that approximately 81% of implants restored with cement‐retained restorations showed cement remnants below the crown margins, often associated with clinical and radiographic signs of inflammation (Wilson Jr. 2009). The risk is increased by the fact that most cement residues are difficult to detect, especially in the case of overcontoured restorations or when the restoration margins are placed submucosally, which can impede complete cement removal and contribute to the development of iatrogenic peri‐implantitis (Pesce et al. 2015; Staubli et al. 2017). Therefore, it is necessary to follow a strict cementation protocol when placing cemented implant restorations to avoid invasion of the cements into the peri‐implant tissues. In this context, the type and amount of cement as well as the cementation technique used have been reported as critical factors in reducing residual cement. Zinc phosphate, zinc polycarboxylate, glass ionomer and resin cements are among the most commonly used materials for the definitive cementation of implant‐supported restorations (Agar et al. 1997; Mehl et al. 2008). A recent in vitro study evaluated the impact of three different cementation techniques and three cement types on the amount of excess cement around implant‐supported crowns (Düzgün et al. 2025). Here, single implant anterior metal crowns with a 1 mm submucosal margin were used. Regardless of the cementation protocol used, the lowest residual cement amount was found in the glass ionomer cement group, whereas the highest amount was observed with resin‐modified glass ionomer cement (Düzgün et al. 2025). With regard to cementation technique, approaches such as pre‐cementing the crown or using crown venting have been shown to influence marginal cement excess and crown marginal quality in implant‐supported CAD/CAM restorations (Zaugg, Meyer, et al. 2018; Zaugg, Zehnder, et al. 2018).

To address these challenges, alternative concepts such as eccentric implant placement combined with narrower, premolar‐shaped crowns and cantilevered pontics have been proposed. An in vitro study demonstrated that this design, featuring a narrow tunnel between the implant and the pontic, significantly improved the removal of simulated biofilm compared to conventional restorations, likely due to a more favorable emergence angle and reduced formation of inaccessible areas (Tuna et al. 2019).

While these design modifications show potential for enhancing hygiene maintenance, their effect on the removal of excess cement remains uncertain. Therefore, the present study aims to evaluate the influence of crown design, cement type, and submucosal margin depth on the efficiency of residual cement removal around single implant‐supported restorations. The null hypothesis states that none of these parameters have a significant effect on the amount of residual cement.

## 2 Materials and Method

Due to the in vitro nature of this study, which was conducted solely on artificial models without the use of human participants or live animals, no ethical approval was required.

### 2.1 Sample Preparation

**FIGURE 1:**
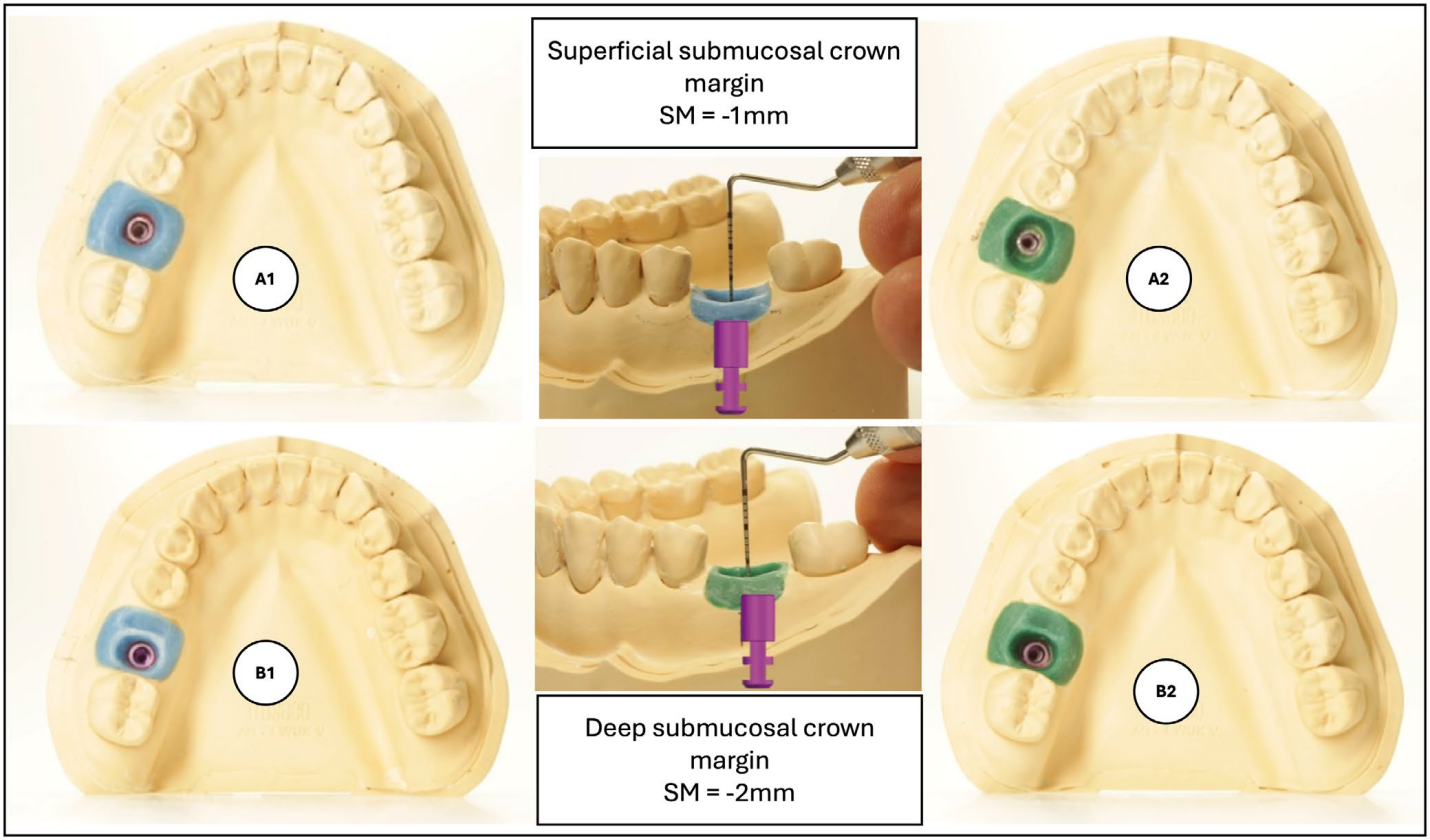
Mandibular gypsum models with a missing lower first molar used for planning the different test groups according to implant position and crown margin depth. A1: Centrally placed implant with a superficial submucosal margin (SM = −1 mm), A2: Centrally placed implant with a deep submucosal margin (DM = −2 mm), B1: Distally placed implant with a superficial submucosal margin (SM = −1 mm) and B2: Distally placed implant with a deep submucosal margin (DM = −2 mm).

Four gypsum mandibular models with a missing lower first molar were fabricated as duplicates from Frasaco models (Frasaco GmbH, Tettnang, Germany). These models were used for planning the different test groups according to implant position and crown margin depth, where A1 and B1 represented a central and distally placed implant, respectively, with a superficial submucosal margin (SM = −1 mm), and A2, B2 represented central and distally placed implants, respectively, with a deep submucosal margin (SM = −2 mm) (Figure 1).

**FIGURE 2:**
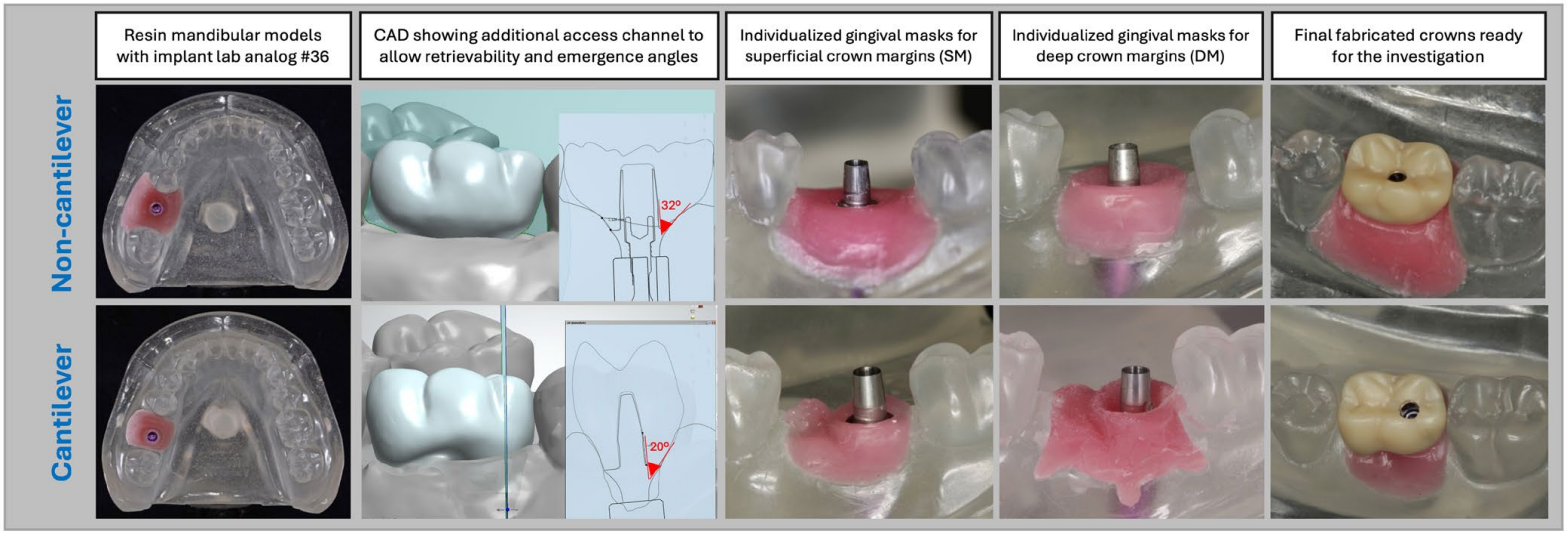
Different steps for restoration fabrication of two different designs (non‐cantilever, cantilever) starting left to right: Mandibular resin models with implant lab analog in region 36, followed by the crown design using a CAD software where crowns were designed with an additional access channel to allow retrievability during the investigation. Note the different emergence angles of the two different crown designs (cantilever = 20°, non‐cantilever = 32°). Individualized gingival masks to create the two different crown margin depths (SM = −1 mm, DM = −2 mm), then the final fabricated non‐cantilever and cantilever crowns ready for the investigation.

After definitive planning, a total of twelve mandibular models (4 per group) were fabricated as duplicates of the original gypsum models using transparent cold‐cured acrylic resin (Palapress, Heraeus Kulzer GmbH and Co. KG, Hanau, Germany). Individual gingival masks were applied to the edentulous region of the lower first molar (#36) using an A‐silicone material (Gingifast Rigid, Zhermack GmbH, Marl, Germany). Implant lab analogs with a diameter of 4.3 mm (Conelog, Camlog Biotechnologies AG, Basel, Switzerland) were placed 4 mm beneath the suppositional cementoenamel junction in two different sagittal positions. Six models received implant analogs centrally in the edentulous gap (in the prosthetic axis of a lower first molar crown), whereas the other six models received implant analogs in a more distal position (in the prosthetic axis of the distal root of a lower first molar). These were further subdivided according to the crown margin depth into superficial and deep submucosal crown margins with crown margin depths of 1 mm and 2 mm, respectively (Figure 2).

In order to simulate non‐individualized abutments, titanium base CAD/CAM abutments (Conelog Titanium base CAD/CAM, Ø4.3 mm, GH = 2 mm, Camlog Biotechnologies AG, Base, Switzerland) were used for the restorations. These were combined with monolithic CAD/CAM‐manufactured zirconia crowns (Zirlux FC2, Henry Schein Dental, Langen, Germany). The crown design differed according to the implant analog position. Centrally placed implant analogs received conventional crowns (non‐cantilever, conventional molar‐shaped crown), whereas the distally placed implant analogs were provided with cantilevered crown designs (a distal premolar crown with a mesial pontic). In the cantilever group, the pontic's basal crown section was root‐shaped, creating an additional interradicular access path for facilitating oral hygiene measures. The different crowns were designed using CAD software and manufactured through a centralized production service (Dedicam, CAMLOG GmbH, Wimsheim, Germany) (Figure 2). A total number of 120 crowns were fabricated and manually polished to a high gloss using goat hairbrushes and a diamond polishing paste (Fegupol Zirkopol, Feguramed GmbH, Buchen, Germany). For the investigation, it was necessary for the cemented crown‐abutment units to be retrievable. Therefore, each crown was modified by incorporating an additional access channel to the abutment screw from the occlusal aspect.

### 2.2 Study Set‐Up

**FIGURE 3:**
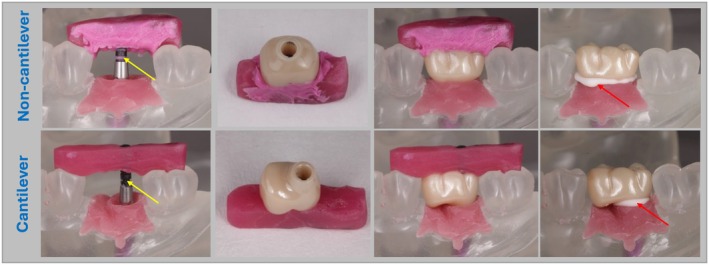
Cementation of the non‐cantilever and cantilever crowns. Yellow arrows indicate the additional access channels specifically designed to facilitate retrievability, temporarily sealed with a resin plug used during cementation to preserve cement pressure and prevent cement from flowing through the occlusal access channels. Red arrows highlight areas of excess cement to be removed by the dentists.

All twelve mandibular models were fixed subsequently in a phantom head (Frasaco GmbH, Tettnang, Germany). The crowns were fixed to the implant abutments using three different cements in a standardized manner and quantity. The types of cements used for the investigation are listed in Table 1. The constant amount of cement was mixed according to the manufacturer's instructions and filled in a standardized manner into the intaglio surface of the crowns till the crown margin. Crowns were then seated onto their corresponding abutment using finger pressure, and a constant force of 50 N was applied axially using special clamps. During cementation, the occlusal access hole was temporarily sealed with a resin plug to maintain cement pressure, simulating clinical conditions (Figure 3).

**TABLE 1:** The different types of cements investigated in the study.

Cement type	Acronym	Name and manufacturer
Glass Ionomer cement	GI	KetacCem, 3 M ESPE, Germany
Polycarboxylate cement	PC	Durelon, 3 M ESPE, Germany
Dual‐curing composite cement	RC	RelyX Unicem 2, 3 M ESPE, Germany

**FIGURE 4:**
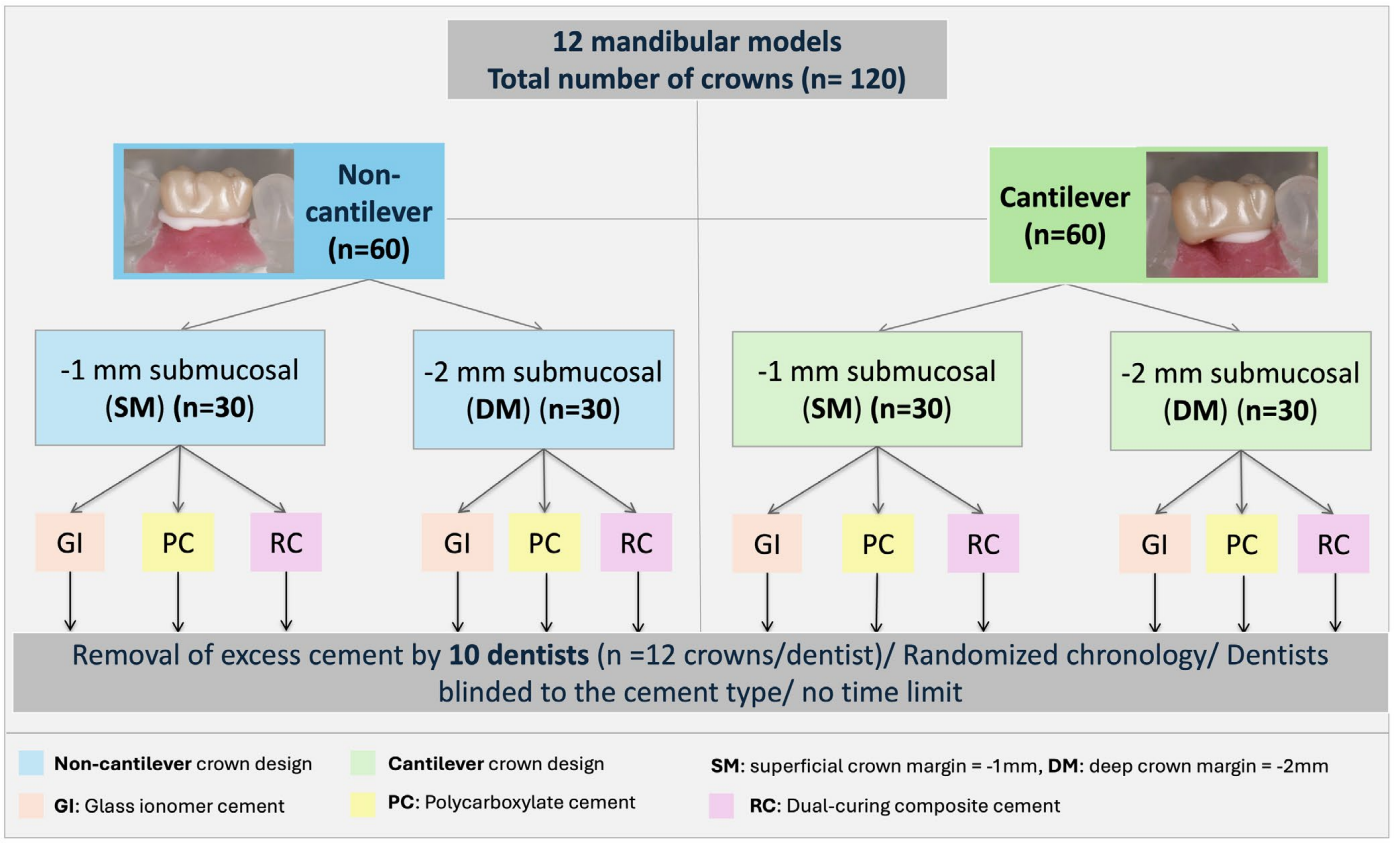
Workflow of the study investigation. A total number of 120 crowns were used for the investigation. Ten dentists were assigned to remove excess cement from margins of two different crown designs (non‐cantilever, cantilever) and two different crown margin depths (SM, DM) cemented with three different cements (GI, PC, RC) in a randomized chronology and with no time limit.

After complete setting of the cement, ten experienced dentists were assigned to remove excess cement from the margins of two different crown designs using rigid metal scalers (HuFriedy SH6/76, Hu‐Friedy Mfg. Co. LLC, Emmingen, Germany) under simulated clinical conditions. Each dentist cleaned one implant‐supported crown for every combination of cement type crown design and margin depth, following a randomized sequence. The time required to remove cement remnants from the abutment surfaces was recorded for each procedure using a stopwatch. After completing the cleaning process, each dentist subjectively assessed whether the cement removal was clinically sufficient. In total, each participant performed twelve cleaning procedures, representing all combinations of crown design, margin depth and cement type (Figure 4).

### 2.3 Study Analysis

Standardized photographs were obtained to visually represent the amount of residual cement. Six cube‐shaped silicone bases with incorporated implant lab analogues were prepared to hold the abutment‐crown samples, which were removed from the models and positioned in the bases. Digital images of the buccal, lingual, mesial, distal, and basal sides of the abutment‐crowns were captured using a custom photographic station, which ensured a constant camera‐to‐object distance, a non‐reflective black background, and secure fixation of the samples.

Quantification of excess cement was performed exclusively by collecting the cement remnants from the crown and abutment surfaces, then weighing it using a high precision scale (Sartorius, AZ214, S.M., USA). Volumetric values were subsequently calculated by converting the measured weights using the known density of each cement.

### 2.4 Statistical Analysis

All statistical analyses were performed using SPSS (IBM SPSS Statistics, Version 29.0.0). A formal a priori power calculation was not performed. The sample size was defined based on the experimental setup and in accordance with similar in vitro studies (Gehrke et al. 2019; Gönder et al. 2023). Effect sizes (partial eta squared, *η* ^2^_p_) were calculated for all main and interaction effects to assess the magnitude of the observed differences. The primary dependent variable was the volume of residual cement (mm^3^). A univariate analysis of variance (UNIANOVA) was initially conducted to evaluate the main and interaction effects of crown design (non‐cantilever vs. cantilever), cement type (GI, PC, RC), and submucosal margin depth (−1 mm vs. −2 mm). Assumptions of normality and homogeneity of variances were assessed using Shapiro–Wilk tests, visual inspection of Q‐Q plots, and Levene's test (Figure S1). As deviations from normality and variance heterogeneity were observed, additional non‐parametric analyses were performed to ensure robust inference. Mann–Whitney *U* tests were used for two‐group comparisons (crown design and margin depth), and a Kruskal–Wallis test was applied for comparisons among cement types, followed by Bonferroni‐corrected post hoc tests when appropriate. The level of statistical significance was set at *p =* 0.05.

## 3 Results

### 3.1 Volume Measurements of Excess Cement

A total of 120 measurements were analyzed to evaluate the influence of crown design, cement type and crown margin depth on the volume of residual cement. The mean residual cement volume ranged between 0.00mm^3^ and 1.19mm^3^ across all groups (Table 2).

**TABLE 2:** Residual cement volumes around investigated implant crowns according to crown design, crown margin depth and cement type. Values are presented as mean ± SD, median and IQR.

Crown design	Crown margin depth	Cement type	Residual cement volume (mm^3^) (mean ± SD)	Median (mm^3^)	IQR (mm^3^)
Non‐cantilever	SM	GI	0.00 ± 0.00	0.00	0.00
		PC	0.19 ± 0.48	0.00^a^	0.00^a^
		RC	0.15 ± 0.25	0.00	0.18
	DM	GI	0.16 ± 0.32	0.00^a^	0.00^a^
		PC	0.83 ± 0.98	0.60	1.35
		RC	1.19 ± 1.83	0.50	1.50
Cantilever	SM	GI	0.00 ± 0.00	0.00	0.00
		PC	0.47 ± 0.99	0.00^a^	0.00^a^
		RC	0.84 ± 0.79	0.45	1.00
	DM	GI	0.14 ± 0.24	0.00	0.23
		PC	0.68 ± 1.52	0.00^a^	0.00^a^
		RC	1.01 ± 1.17	0.65	1.70

Abbreviations: DM, deep crown margin; Gi, glass ionomer cement; IQR, interquartile range; PC, polycarboxylate cement; RC, Dual‐curing composite cement; SD, standard deviation; SM, superficial crown margin.

^a^ Indicates that ≥ 50% of measurements were zero; median and IQR are therefore 0.00, while mean ± SD reflect the few non‐zero values.

The univariate analysis of variance revealed a significant effect of cement type on residual cement volume (*p* = 0.003, *η* ^2^_p_ = 0.105), indicating a moderate effect size. Crowns cemented with RC generally exhibited the highest residual cement volumes, while GI showed the lowest. The mean residual cement volume for GI, PC, and RC was 0.07 ± 0.20 mm^3^, 0.54 ± 1.04 mm^3^ and 0.79 ± 1.18 mm^3^, respectively. Post hoc comparisons revealed statistically significant differences between GI and RC (*p* < *0.001*) and between PC and RC (*p* = *0.022*).

A significant effect of margin depth was also observed (*p* < *0.001*, *η* ^2^_p_ = 0.048), corresponding to a small to moderate effect size. Deep margins (DM) were associated with higher residual cement volumes (0.67 ± 1.19 mm^3^) compared to superficial margins (SM) (0.28 ± 0.62 mm^3^).

Regarding crown design, both non‐cantilever and cantilever crowns cemented with GI resulted in the lowest mean residual cement volumes, regardless of crown margin depth. A slight tendency toward lower residual cement values was observed in cantilever crowns, particularly under deep margin conditions; however, this trend was not consistent across all cement types and did not reach statistical significance (*p* = 0.558, *η* ^2^_p_ = 0.004), indicating a negligible effect size.

**FIGURE 5:**
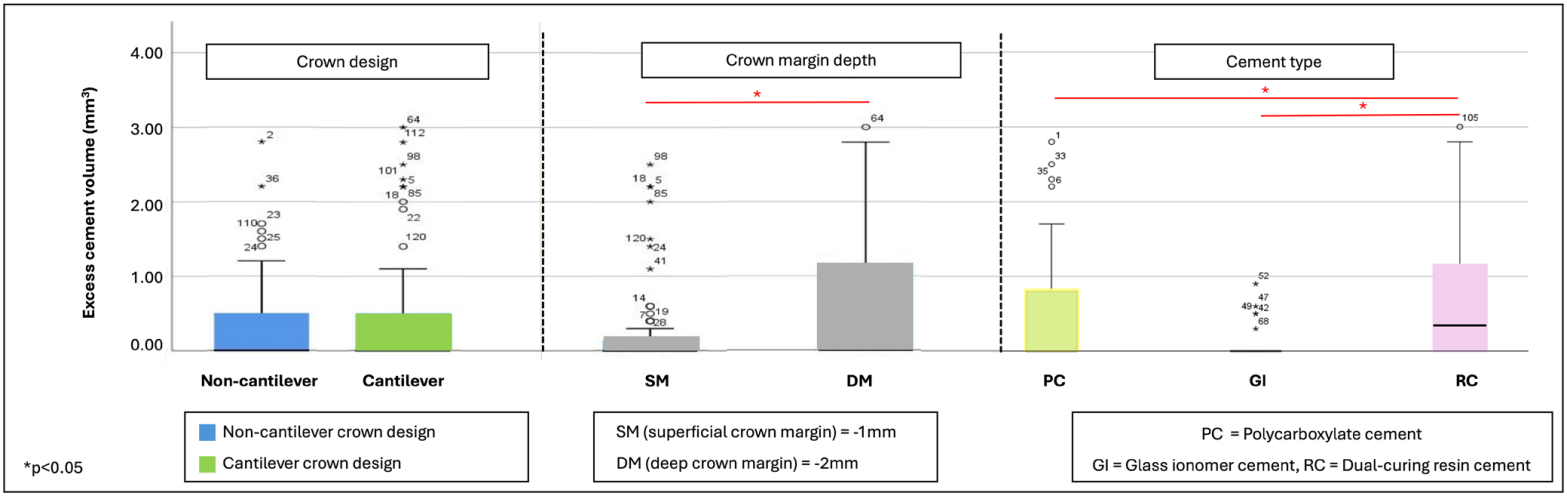
Boxplots representing median and IQR of the excess cement volume of the different crown designs with the two investigated crown margin depths (SM = −1 mm, DM = −2 mm), regardless of cement type used (left and middle). Right boxplot represents the excess cement volume of the different cements regardless of crown design and crown margin depth. Red line represents significant differences between the groups.

Given the non‐normal distribution of the data, results are presented as medians and interquartile ranges, which correspond to the boxplots shown in Figure 5.

**FIGURE 6:**
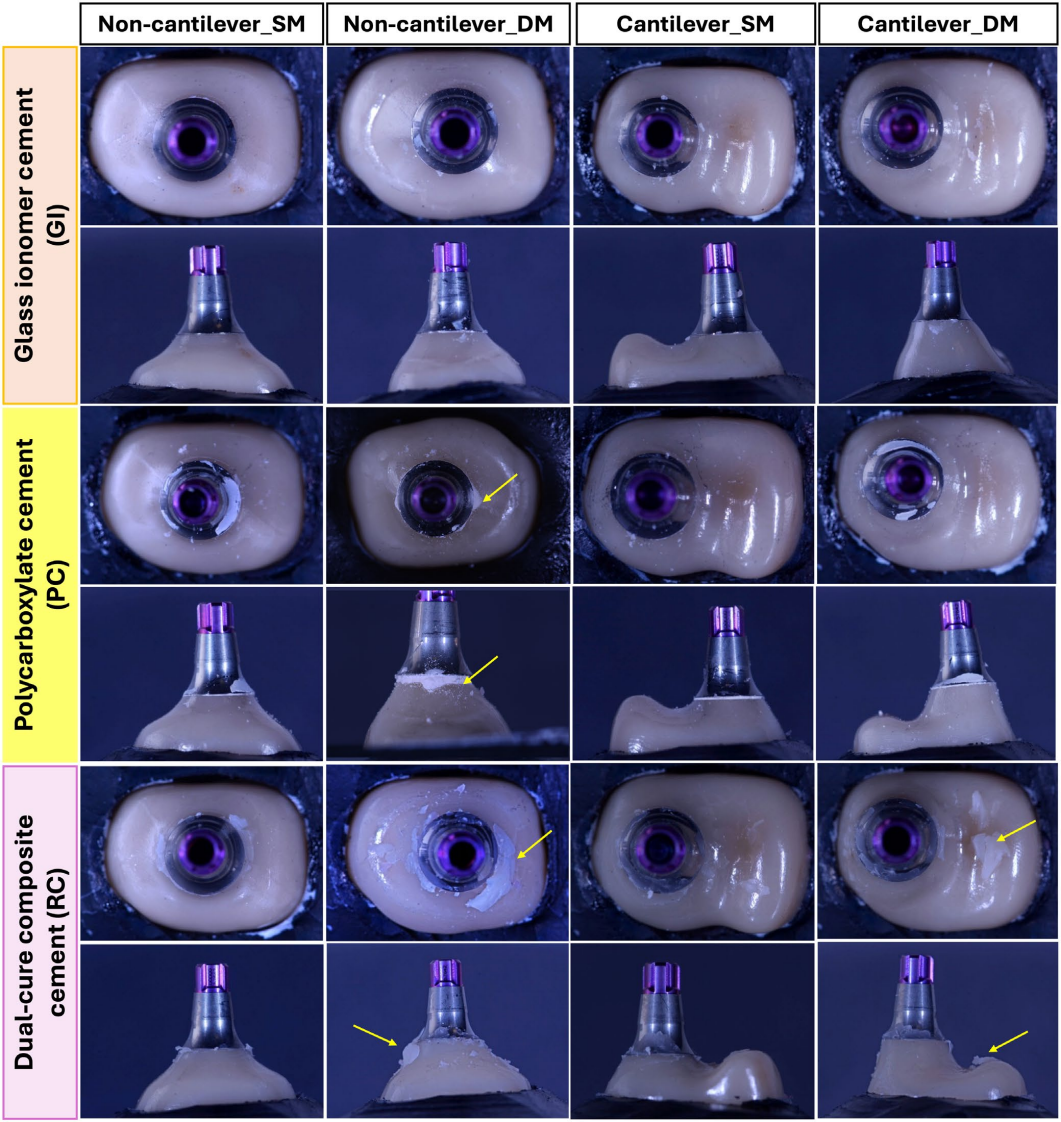
Standardized digital images illustrating residual cement after removal by clinicians across various crown designs (non‐cantilever, cantilever), margin depths (SM, DM), and cement types (GI, PC, RC). Yellow arrows indicate visible cement remnants on different surfaces of the crown restorations.

The photographic images presented in Figure 6 illustrate the investigated implant‐supported crowns from various aspects, including the mesial, distal, buccal, oral, and basal surfaces. A notable accumulation of cement remnants was consistently observed on the distal and basal surfaces, particularly in crowns with deeper submucosal margins (DM), irrespective of the cement type used. However, crowns cemented with resin cement (RC) exhibited a tendency for greater amounts of residual cement compared to other types.

### 3.2 Time Analysis

**FIGURE 7:**
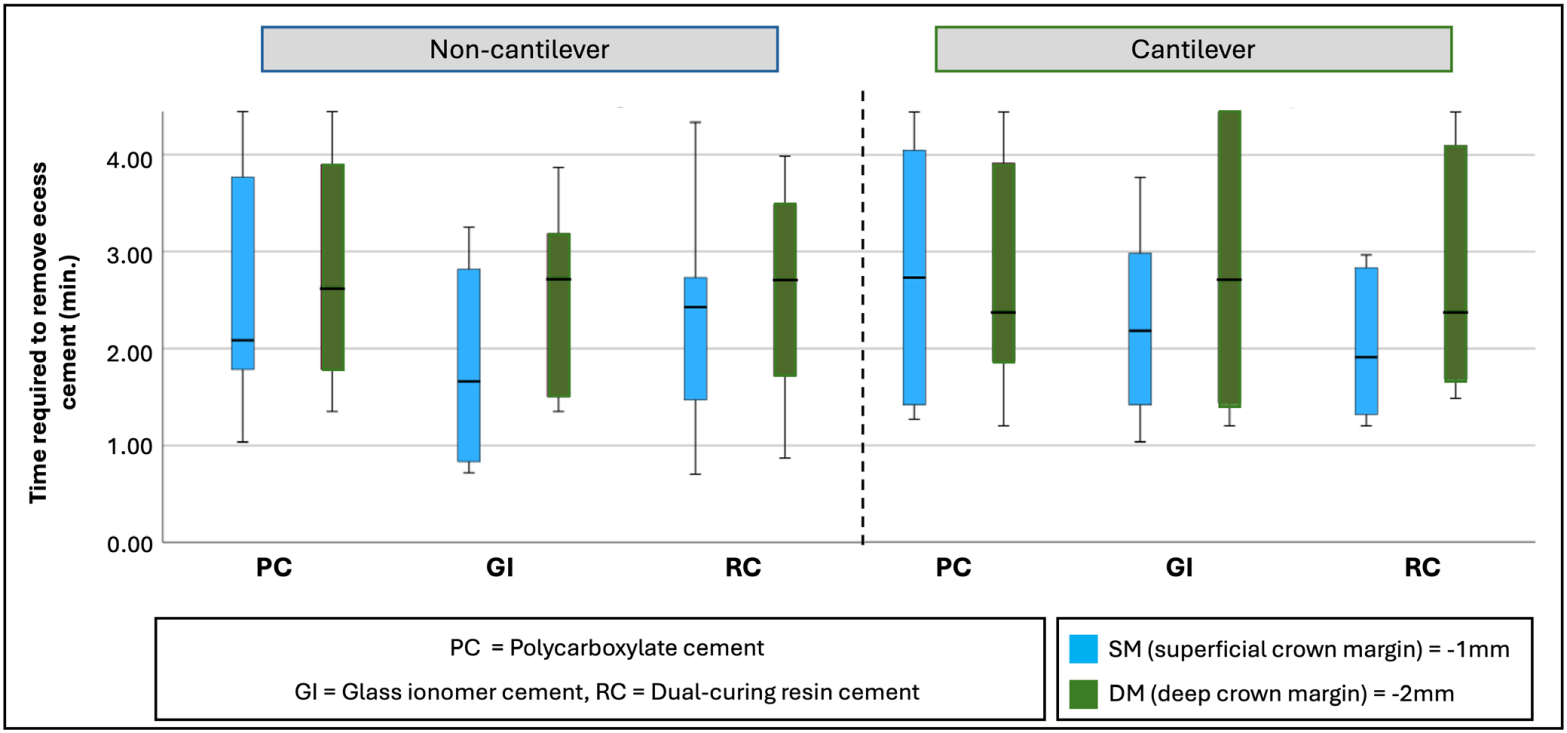
Boxplots representing the time required to remove the excess of the three investigated cements around crowns with different design and margin depths. No statistical significance between the three different cements could be found.

The time required to remove the excess cement was recorded for each restoration by all participants. The recorded removal times ranged from a minimum mean value of 1.78 ± 0.92 min for the group non‐cantilever_SM_GI and a maximum mean value of 3.03 ± 1.46 min for the group non‐cantilever_DM_PC (Table 3). Statistical analysis revealed no significant differences in removal time between the different groups (*p* > *0.05*), indicating that none of the evaluated variables had a measurable impact on the duration required for cement (Figure 7).

**TABLE 3:** Mean values with standard deviations for the time required to remove the excess cement according to crown design, crown margin depth, and cement type.

Crown design	Crown margin depth	Cement type	Time required to remove excess cement (min.) (mean ± SD)
Non‐cantilever	SM	GI	1.78 ± 0.92
		PC	2.83 ± 1.75
		RC	2.49 ± 1.29
	DM	GI	2.73 ± 1.36
		PC	3.03 ± 1.46
		RC	2.86 ± 1.77
Cantilever	SM	GI	2.26 ± 1.00
		PC	2.86 ± 1.93
		RC	2.28 ± 1.31
	DM	GI	2.91 ± 1.55
		PC	2.85 ± 1.45
		RC	3.01 ± 1.69

Abbreviations: DM, deep crown margin; Gi, glass ionomer cement; min, minutes; PC, polycarboxylate cement; RC, dual‐curing composite cement; SD, standard deviation; SM, superficial crown margin.

## 4 Discussion

The current in vitro study evaluated the influence of crown design, margin depth, and cement type on cement removal around single implant‐supported restorations. Among these factors, crown margin depth had a statistically significant effect on residual cement, highlighting its critical role in prosthetic planning and peri‐implant health.

The *design of the implant crown*, whether centrally or distally positioned, showed a variable influence on the amount of residual cement. Non‐cantilever crowns exhibited slightly higher and more variable residual cement volumes, particularly with deeper crown margins. In contrast, cantilever designs demonstrated more consistent and generally lower residual cement volumes with all cement types. While the crown design alone did not yield statistically significant differences, the interaction with cement type suggests that crowns with cantilevers and deep crown margins may facilitate more effective cement removal. This may be attributed to the restoration geometry, as non‐cantilever crowns typically involve larger emergence angles and more convex profiles, both of which have been previously associated with increased difficulty in cement removal and reduced accessibility for oral hygiene measures (Park et al. 2022; Tuna et al. 2019). These findings are consistent with clinical and radiographic studies indicating that prosthetic designs with convex emergence profiles and emergence angles exceeding 30° are associated with increased marginal bone loss and unfavorable peri‐implant tissue outcomes (Katafuchi et al. 2018; Misch et al. 2025; Strauss et al. 2022). Furthermore, previous systematic reviews have shown that implant restorations with emergence angles ≤ 30° and platform‐matched designs may have a positive effect on marginal bone stability, especially when convex profiles are avoided (Atieh et al. 2023; Izzetti et al. 2025).

With regard to *crown margin depth*, deeper crown margins were associated with significantly greater residual cement volumes, regardless of the cement type or crown design. This finding aligns with prior studies suggesting that submucosal margins hinder visibility and mechanical access during cement removal, increasing the risk of leaving behind cement remnants (Gehrke et al. 2019; Staubli et al. 2017). In a similar study investigating the influence of emergence profile and crown margin depth on the amount of undetected cement excess around cement‐implant supported crowns in the anterior region, significantly more cement remnants were detected as the crown‐abutment margin was located more submucosally for each tested restoration (Sancho‐Puchades et al. 2017). Clinically, this supports the recommendation to maintain crown margins as coronally as feasible, or slightly below the gingival margin, to facilitate thorough cement removal and reduce the risk of peri‐implantitis (Staubli et al. 2017). Notably, in the present study, almost no residual cement was detected in either crown design when the crown margins were superficial, particularly when glass ionomer cement was used. This reinforces the notion that margin depth is a more critical determinant of cement retention than crown geometry, and that superficial margin placement should be prioritized whenever clinically feasible to enhance cleanability and reduce biological complications associated with cement excess.

Similar to several previous studies (Düzgün et al. 2025; Gönder et al. 2023; Kuşçu and Hayran 2025; Yıldız et al. 2024), the *type of cement* used had a statistically significant effect on cement removal efficiency. Glass ionomer cement (GI) exhibited the lowest residual cement volumes across both crown designs, particularly in the superficial crown margin groups (SM), where no detectable cement remnants were observed for either the non‐cantilever or cantilever restorations. In contrast, polycarboxylate cement (PC) and resin cement (RC) were associated with higher but more consistent residual volumes, especially when used in conjunction with deep margins (DM). Overall, RC demonstrated the highest residual cement values among all the investigated materials, making it the least favorable in terms of excess cement management. However, these findings should be interpreted with caution, as cement removal in the current in vitro study was initiated only after complete setting of the material. This contrasts with the manufacturer's instructions, which recommend excess removal following a brief light‐curing (tack‐curing) or during the gel phase, when the cement is more easily retrievable. Due to the standardized protocol applied across all cement types in this study, and the constraints of the in vitro setup, early‐stage removal as recommended by the manufacturer was not feasible. This methodological choice, though necessary for experimental consistency, may have disproportionately affected the performance of RC in comparison to other cements.

Although cement properties such as viscosity, working time, and opacity affect clinical handling, margin depth appears to be the primary determinant of residual cement retention, with deeper margins hindering cement removal. These findings support the use of glass ionomer cement when supragingival margins are feasible and emphasize that cement selection should be considered in relation to margin depth and prosthetic design. Additionally, cementation technique and isolation significantly influence residual cement around implant‐supported restorations (Düzgün et al. 2025; Kuşçu and Hayran 2025).

Although the influence of crown margin depth, cement type, and prosthetic design on residual cement has been widely studied, posterior implant‐supported cantilevered crowns have rarely been evaluated. The present findings suggest that prosthetic geometry may affect cement removal, as cantilevered crowns exhibited lower and more consistent residual cement levels than non‐cantilever designs, albeit without statistical significance. This trend may be clinically relevant, as the reduced emergence profile of cantilevered crowns could facilitate cement removal and hygiene access, supporting peri‐implant tissue maintenance.

Although this study was designed to simulate real‐life clinical conditions, its in vitro nature by working on artificial dental models mounted on a phantom unit presents several inherent limitations. Controlled laboratory settings do not fully capture the complexities of the oral environment, including variables such as soft tissue resilience, bleeding, salivary flow, or operator variability. The artificial gingiva used in the model lacked the elastic and anatomical characteristics of natural peri‐implant soft tissues, which may have influenced both the seating of the restorations and the ease of cement removal. Moreover, while the viscosity properties of the different cements were acknowledged as a factor potentially affecting complete seating and residual cement, no intraoral fluid simulation was incorporated, limiting the ability to assess how these materials would behave under actual clinical conditions.

The sample size used may have limited the ability to detect very small effects, particularly for interaction terms. However, the calculated effect sizes indicated that interaction effects were consistently small (*η* ^2^_p_ ≤ 0.016), suggesting limited practical relevance. In contrast, moderate effects were observed for cement type and margin depth, which were successfully detected. Therefore, while the possibility of undetected small effects cannot be excluded, the main findings of this study can be considered robust.

It is also important to mention that in posterior mandibular regions and non‐esthetic areas, screw‐retained restorations and tissue‐level implants are generally recommended, as they facilitate maintenance and eliminate the risk of cement‐related biological complications. Current consensus statements and systematic reviews indicate that cement‐retained restorations, particularly on bone‐level implants, are associated with an increased risk of peri‐implant disease when excess cement cannot be adequately controlled (Fiorillo et al. 2024; Wittneben et al. 2014). Nevertheless, cement‐retained restorations on bone‐level implants remain a treatment option, which is still encountered in daily practice. When cementation is required, the use of individualized abutments with controlled, accessible cement margins has been shown to substantially reduce the risk of residual cement and related biological complications (Greenstein et al. 2025; Kim et al. 2023). Therefore, while a tissue‐level implant may be preferable in many posterior mandibular cases, the implant–prosthetic concept used in the present study reflects a realistic clinical scenario within the spectrum of established implant prosthodontic approaches.

## 5 Conclusions

Within the limitations of this in vitro study, deeper submucosal crown margins were consistently associated with higher volumes of residual cement, identifying margin depth as the most critical factor in cement removal efficacy. This finding emphasizes the clinical importance of placing crown margins at or slightly below the mucosal margin. Although cement type influenced residual cement, with glass ionomer showing consistently lower values, crown design showed no statistically significant effect and did not demonstrate a consistent advantage.

## Author Contributions

T.T., S.W. and S.B. conceived, designed and planned the study, C.A., S.B. and T.T. carried out the experiments, C.A., A.R. and S.B. performed the analytical calculations and statistical analysis, S.B. and T.T. contributed to the interpretation of the results, S.B. and T.T. wrote the manuscript with input from all authors (C.A., A.R. and S.W.), S.W. and T.T. supervised the whole project. S.W., T.T. and S.B. edited and revised the final manuscript.

## Funding

This research was funded by a grant from CAMLOG Foundation (Reference number: CF21402).

## Ethics Statement

Ethics approval was not required for this in vitro study.

## Conflicts of Interest

The authors declare no conflicts of interest.

## Supporting information


**Figure S1:** Q–Q plots of volume measurements (mm^3^) stratified by crown design (CCD and ACD). Both plots show clear deviations from normality, supporting the reporting of medians and interquartile ranges in addition to means and standard deviations.

**Data S1:** CRIS Guidelines (Checklist for reporting in vitro studies).

## Data Availability

The data that support the findings of this study are available from the corresponding author upon reasonable request.
