# Supplementary figures and images for: Effect of Crown Design, Cement Type and Margin Depth on the Removal of Cement Remnants Around Single Implant‐Supported Restorations. An In‐Vitro Study
Source: Clin Oral Implants Res. 2026 Apr 12;37(7):856–66. doi: 10.1111/clr.70129 (PMC13340517; doi:10.1111/clr.70129)

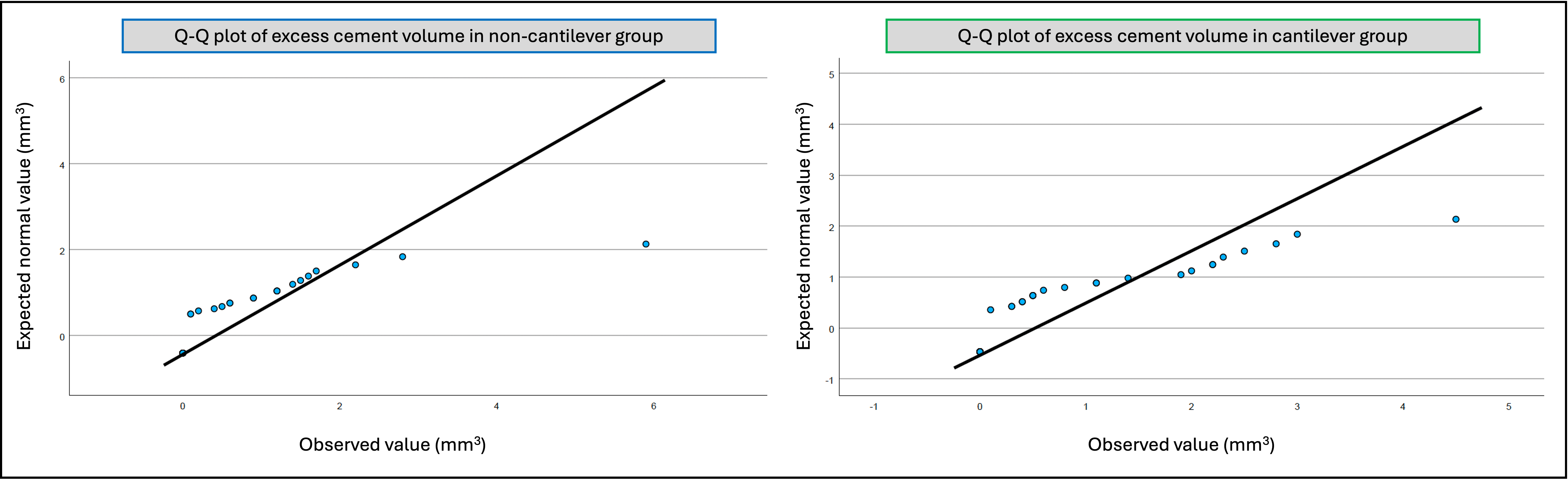

Supplement: Supplementary file 1 — Figure S1: Q–Q plots of volume measurements (mm3) stratified by crown design (CCD and ACD). Both plots show clear deviations from normality, supporting the reporting of medians and interquartile ranges in addition to means and standard deviations. [file CLR-37-856-s002.tiff]
