# Supplementary material for: Effect of Crown Design, Cement Type and Margin Depth on the Removal of Cement Remnants Around Single Implant‐Supported Restorations. An In‐Vitro Study
Source: Clin Oral Implants Res. 2026 Apr 12;37(7):856–66. doi: 10.1111/clr.70129 (PMC13340517; doi:10.1111/clr.70129)
Supplement: Supplementary file 2 — Data S1: CRIS Guidelines (Checklist for reporting in vitro studies). [file CLR-37-856-s001.docx]

**CRIS Guidelines (Checklist for reporting in-vitro studies)**

| **Section/Topic** | **Checklist item** | **Reported on page number** |
| --- | --- | --- |
| **Title and abstract** |  |  |
|  | Identification as an in-vitro/laboratory study in the title | 1 |
|  | Structured summary of trial design, methods, results, and conclusions | 2 |
| **Introduction** |  |  |
| Background and objectives | Scientific background and explanation of rationale | 3-5 |
|  | Specific objectives or hypotheses | 5 |
| **Methods** |  |  |
| Interventions | The intervention for each group, including how and when they were actually administered, with sufficient detail to allow replication | 6-8 |
| Outcomes | Completely defined pre-specified primary and secondary outcome measures, including how and when they were assessed | 6-8 |
| Sample size | How sample size was determined | 7 |
| Randomisation:   - Sequence generation - Allocation concealment mechanism - Implementation | - Method used to generate the random allocation sequence - Mechanism used to implement the random allocation sequence, describing any steps taken to conceal the sequence until interventions were assigned - Who generated the random allocation sequence, and who assigned samples to intervention | NA |
| Blinding | If done, who was blinded after assignment to interventions, and how | NA |
| Statistical methods | Statistical methods used to compare groups for primary and secondary outcomes | 8,9 |
| **Results** |  |  |
| Numbers analysed | For each group, number of items included in each analysis and whether the analysis was by original assigned groups | 9 |
| Outcomes and estimation | For each outcome, results for each group, and the estimated effect size and its precision (such as 95% confidence intervals | 9-10 |
| **Discussion** |  |  |
| Limitations | Trial limitations, addressing sources of potential bias, imprecision, and, if relevant, multiplicity of analyses | 14 |
| Generalisability | Generalisability (external validity, applicability) of the trial findings | 10-15 |
| Interpretation | Interpretation consistent with results, balancing benefits and harms, and considering other relevant evidence | 14,15 |
| **Other information** |  |  |
| Protocol | Where the full trial protocol can be accessed, if available | Title page |
| Funding | Sources of funding and other support, role of funders | Title page |
